# Analyzing the Total Attractive Force and Hydrogen Storage on Two-Dimensional MoP2 at Different Temperatures Using a First-Principles Molecular Dynamics Approach

**DOI:** 10.3390/molecules29225228

**Published:** 2024-11-05

**Authors:** Alma Lorena Marcos Viquez, Osiris Salas Torres, Luis Fernando Magaña Solís

**Affiliations:** 1 Instituto de Física, Universidad Nacional Autónoma de México, Apartado Postal 20-364, Mexico City 01000, Mexico; almalorenamarcos@gmail.com; 2 Instituto Politécnico Nacional, Escuela Superior de Ingeniería Mecánica y Eléctrica, Avenida Instituto Politécnico Nacional, S/N, Mexico City 07340, Mexico; kaled_o7@yahoo.com.mx

**Keywords:** 2D materials, 2D MoP_2_, surface forces, adsorption

## Abstract

We performed first-principle molecular dynamics (FPMD) calculations to test the total attraction force on a physisorbed molecule at a given temperature and ambient pressure and applied it to the hydrogen storage on the 2D material MoP2. We considered a pristine material and one with 12.5% of Mo vacancies. By optimization, we calculated a gravimetric capacity for pristine MoP2 of 5.72%, with an adsorption energy of −0.13 eV/molecule. We found 6.02% and −0.14 eV/molecule for the defective surface. Next, we applied our approach to determine if the molecular hydrogen physisorption obtained by simple energy optimization exists for a given temperature and ambient pressure. We used this approach to determine the number of molecules adsorbed on the surface at a given temperature. Thus, we conducted a FPMD calculation at temperature T_1_, using optimization as the initial system configuration. Subsequently, we performed a second FPMD calculation at a temperature T_2_ (with T_2_ << T_1_), using the steady configuration of the first FPMD calculation as the initial configuration. We identified as adsorbed molecules at temperature T_1,_ only those forced back toward the surface at temperature T_2_ due to kinetic energy loss at the lower temperature. The defective surface gave the best gravimetric capacity, ranging from 5.27% at 300 K to 6.02% at 77 K. The latter met the requirement from the US-DOE, indicating the potential practical application of our research in hydrogen storage.

## 1. Introduction

Scientific interest in finding alternatives to the worldwide problems stemming from population growth and limited fossil fuel resources has increased in recent years. The focus has been on producing green energy and using materials to store it. Hydrogen storage is a desirable option for this purpose.

The development of materials for hydrogen storage remains a significant challenge. The U.S. Department of Energy (US-DOE) highlights the importance of finding a material to store hydrogen with useful gravimetric and volumetric density that is environmentally friendly [1]. To achieve optimal adsorption and desorption, the adsorption energies for storing H_2_ molecules should fall within the range of 0.1 eV to 0.2 eV [2,3]. Various methods exist to generate renewable energy sources for hydrogen production. Electrolytic hydrogen production offers an alternative to internal combustion engines and fuel cells [4]. The hydrogen evolution reaction (HER) is a critical electrocatalytic reaction, with hydrogen adsorption on the electrode playing a crucial role. In the past, Pt-based materials were considered the best HER catalysts. However, it is essential to find cheaper and more abundant alternatives to Pt materials [5]. Molybdenum sulfide was the first reported alternative to Pt-based materials for catalyzing the HER in acidic aqueous solutions [6]. Since then, research on low-cost HER electro-catalysis materials has gained prominence [7,8,9,10,11,12,13,14].

A current field of investigation is searching for adequate materials for molecular hydrogen storage with the necessary gravimetric capacity at ambient temperature and pressure [15,16,17,18,19,20,21,22,23,24,25,26,27,28,29,30,31,32]. The research interest in hydrogen energy is based on its great potential to replace fossil fuels [33].

On the other hand, currently, the first-principles calculations applied in the exploration of new materials for hydrogen storage are performed solely by simple energy optimization, as seen in [23,33,34]. Our aim in this investigation is to have a way to predict if the molecular physisorption obtained by simple energy optimization exists for a given temperature. This means determining if the kinetic energy given to the system when the effect of temperature is included, destroys the molecular physisorption obtained by simple energy optimization.

Notice that physisorption is a physical adsorption in an exothermic process. In this case, the interaction of the molecule and the surface is dominated by van der Waals forces. The molecular adsorption on a surface is strongly related to electromagnetic interactions. It is usual to consider the image forces in these interactions. The image forces may create image-bound states around small clusters [35,36]. It is normal to associate van der Waals forces with three interactions. The first refers to those between two permanent dipoles; the second refers to the force between a permanent dipole and the corresponding induced dipole. They are the van der Waals interactions’ static (zero frequency) part. They are coulomb interactions already considered in the DFT equations.

The third is the force between two instantaneous and high-frequency oscillating induced dipoles originating from quantum fluctuations. The last one is known as the London dispersion force. Usually, in condensed matter physics, the London dispersion force is called the Van der Waals force. It has a non-classical origin, and we utilize this definition in this work. The London dispersion force is a weak intermolecular interaction from the quantum-induced instantaneous polarization multipoles in molecules [37,38]. Non-polar molecules confirm London forces are essential in the interaction between molecules and surfaces. The interaction decreases with increasing distance. The energy involved in this interaction is low, around 0.2 eV per molecule. Thus, since these are weak interactions, calculating physisorption processes requires accurate methods.

We investigated the direct interaction between a MoP_2_ surface and H_2_ molecules for hydrogen storage using DFT calculations. Additionally, we explored the influence of Mo vacancies on the gravimetric capacity of hydrogen adsorption on a MoP_2_ surface. Our study involved FPMD calculations at atmospheric pressure and 300 K, 77 K, 4 K, and 3 K to assess the system’s stability under varying conditions.

## 2. Results

### 2.1. Hydrogen Storage on Pristine 2D MoP_2_

#### 2.1.1. Optimization

**Figure 1:**
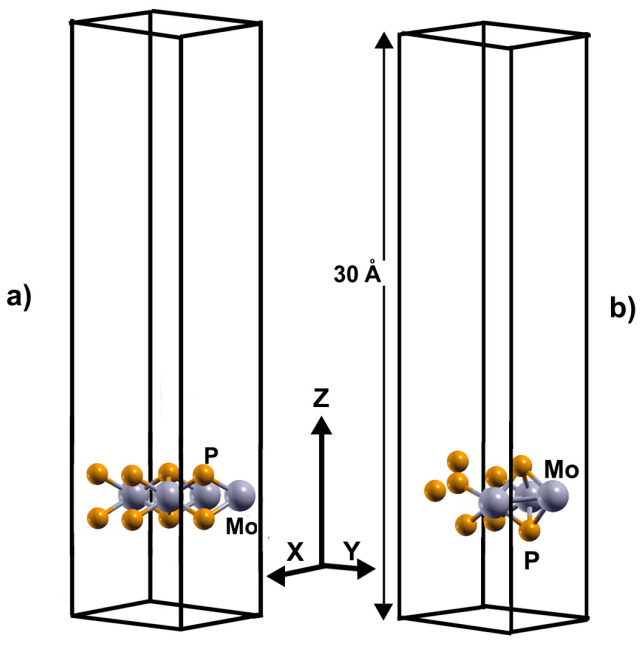
The supercell of the optimized configurations of 2DMoP_2_. In (**a**) for the pristine surface and (**b**) the supercell for the surface with a Mo vacancy.

We simulated these systems with a supercell 2 × 2 and a separation between layers of 30 Å. In the case of the pristine 2DMoP_2_ system, the supercell consists of four molybdenum (Mo) atoms and eight phosphorus (P) atoms (Figure 1a). In comparison, the 2D MoP_2_ with a Mo vacancy has three molybdenum (Mo) particles and eight phosphorus (P) atoms (Figure 1b). In this manner, the Mo vacancy in the cell of twelve atoms corresponds to 12.5% of vacancies. In the system’s initial configuration (pristine or with 12.5% of vacancies), we added hydrogen molecules one by one above a Mo atom (or vacancy) or P atom before the optimization of the system, and the original distance of the hydrogen molecules from the surface was 3 Å.

This distance is large enough to avoid an artificial initial electron exchange with the atoms on the surface. Furthermore, the distance should not be too large because the long-range interaction with the surface must be strong enough to produce attraction on the molecule. Given that the hydrogen molecule size is around 1 Å, and the radiuses of the atoms on the surface are about the same magnitude, an initial distance from the hydrogen molecule to the surface of 3 Å is large enough to avoid this initial electron exchange and adequate to have an adsorption process.

**Figure 2:**
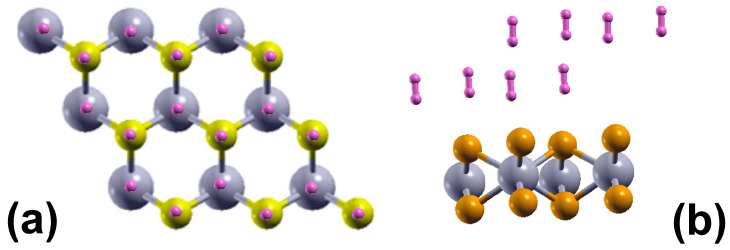
In (**a**), we show the system’s initial configuration from above; we added hydrogen molecules one by one above a Mo or P atom, and the original distance of the hydrogen molecules from the surface was 3 Å before optimizing the system. In (**b**), we have the optimized configuration of the 2DMoP_2_ pristine surface with eight adsorbed hydrogen molecules. This occurs on each face of the material. Thus, the surface adsorbed sixteen molecules, which means *n* = 16 in Equation (2), and a gravimetric capacity of 5.472%. The average adsorption energy is −0.13 eV/molecule.

The pristine monolayer can adsorb up to sixteen H_2_ molecules—eight molecules per face of the 2D material, as shown in Figure 2, which presents the initial and final configurations of the system for the upper face of the surface. Using Equation (1), with *n* = 16, we obtained an average adsorption energy of −0.13 eV/molecule. From Equation (2) and the same value for *n*, we got a gravimetric capacity for hydrogen storage of 5.472%.

#### 2.1.2. First-Principle Molecular Dynamics Calculations

We followed the criterion described in Section 4 to calculate the gravimetric capacity, determining how many molecules adsorbed the surface at a given temperature. Using the optimization of the system and the adsorbed hydrogen molecules as the starting point, we performed the first-principles molecular dynamics calculations at 300 K. We determined the equilibration time at each temperature long enough to obtain a steady system configuration. In our case, it is ~1 ps, which is a typical time to reach a steady configuration when using FPMD simulations see, for example [39]. When this situation is reached, the energy oscillations are regular, around the same energy value, and stay like that afterward.

**Figure 3:**
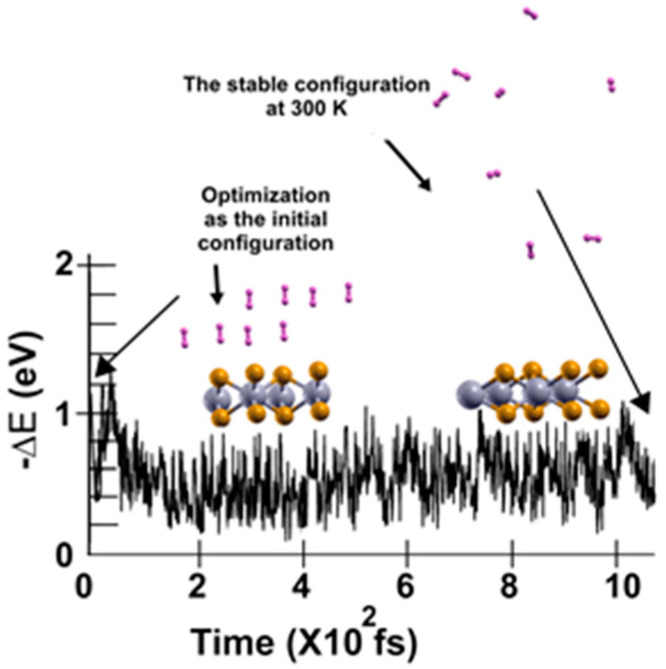
The FPMD calculation is at 300 K. The initial configuration is the optimization of the pristine 2D MoP_2_. After one picosecond, the system reached a steady configuration, oscillating around the same value in −∆E. All the H_2_ molecules moved away from the surface. We diminished the system’s temperature to determine how many molecules remained bound to the surface, as shown in Figure 4.

We found that at 300 K, all the adsorbed H_2_ molecules in the initial optimization moved away from the surface, as Figure 3 shows.

**Figure 4:**
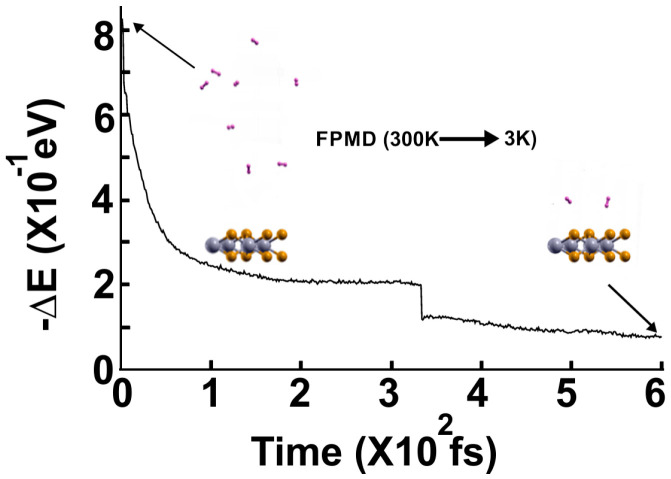
We performed the FPMD calculation at 3 K, taking the initial configuration of the steady system as obtained at 300 K. After 600 femtoseconds, the system reached a steady configuration. We noticed that only two hydrogen molecules got closer to the surface; they moved back to the surface. The remaining six did not change the distances from the surface they had at 300 K, indicating that only those two remained adsorbed on the pristine 2D MoP_2_ surface at 300 K. In this case, the gravimetric capacity is 1.28%.

We then reduced the temperature to 3 K and performed a second FPMD calculation to determine how many remained bound to the surface. Thus, we considered the steady configuration obtained at 300 K as the initial state for the second FPMD calculation, as shown in Figure 4. After reaching a steady configuration (at ~600 fs), we can see that only two hydrogen molecules got closer to the surface. The remaining six did not change the distances from the surface they had at 300 K, indicating that only those two remained adsorbed on the pristine 2D MoP2 surface at 300 K. We identified as adsorbed H_2_ molecules, only those that moved back to the surface. In this way, at 300 K, the pristine surface retained only two H_2_ molecules per face of the material. Thus, taking *n* = 4 in Equation (2), we obtained a gravimetric storage capacity of 1.28%.

**Figure 5:**
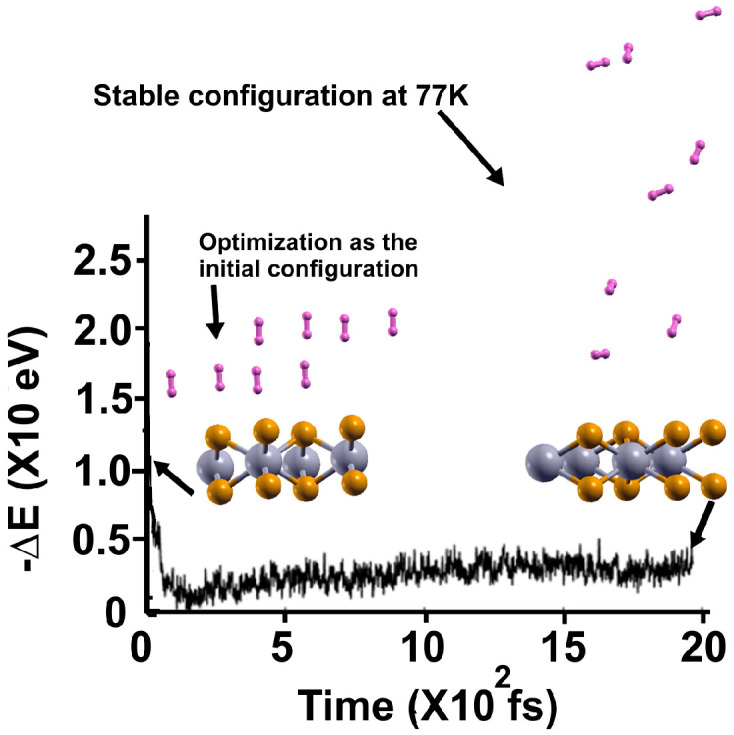
The FPMD calculation is at 77 K, with the optimization as the initial configuration. After two picoseconds, the system reached a steady configuration, oscillating around the same value in −∆E. All the H_2_ molecules are displaced away from the surface. Afterward, we diminished the system’s temperature from 77 K to 3 K to determine how many molecules remained bound to the surface at 77 K, as shown in this figure, to calculate the gravimetric capacity at 77 K from Equation (2).

**Figure 6:**
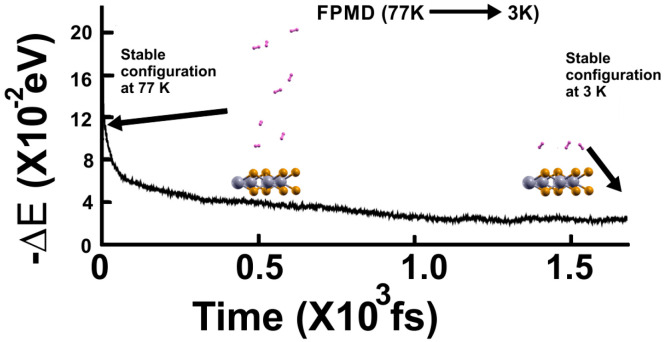
The FPMD calculation is at 3 K, taking the initial configuration of the steady system configuration as obtained at 77 K. The system reached a steady configuration after 1.5 picoseconds. Thus, at 3 K, only three hydrogen molecules (per face of the material) moved back to the surface, indicating that only six remained adsorbed on the pristine 2D MoP_2_ surface at 77 K. Using *n* = 6 in Equation (1), we obtained a gravimetric capacity of 1.92%.

Again, at 77 K, we considered the optimization configuration the initial state and performed an FPMD calculation, as shown in Figure 5, and used the same criterion. Afterward, we reduced the temperature to 3 K and performed another FPMD calculation to determine how many remained bound to the surface at 77 K (see Figure 6). Thus, we identified as adsorbed H_2_ molecules at 77 K, only those that moved back to the surface. We notice that only three H_2_ molecules per face of the material moved back to the surface. Thus, *n* = 6. Using Equation (2), we obtained a gravimetric capacity for molecular hydrogen storage at 77 K of 1.92%.

**Figure 7:**
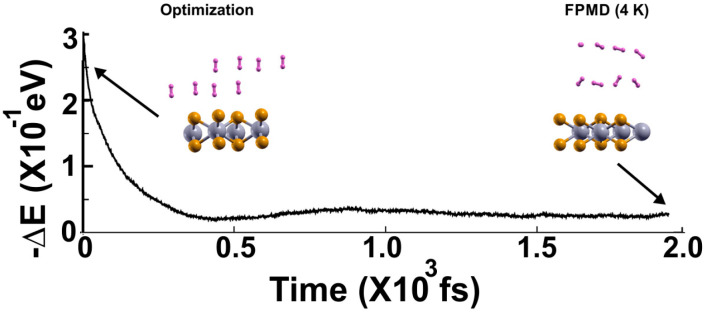
The FPMD calculation is at 4 K, with the optimization as the initial configuration. The system reaches a steady configuration at around two picoseconds. Notice that the eight hydrogen molecules are displaced away from the surface much less than in the other two previous cases, at 300 K and 77 K. Afterward, we diminished the system’s temperature from 4 K to 3 K to determine how many molecules are moved back to the surface from their average positions in the steady configuration at 4 K, as shown in Figure 8, to calculate the gravimetric capacity at 4 K using Equation (2).

In the case of 4 K, Figure 7 shows the FPMD calculation, with the optimization as the initial configuration. After around two picoseconds, the system reaches a steady configuration for 4 K. Notice that all the molecules remain much closer to the surface than in the other two cases. Still, the molecules are displaced away from the surface.

**Figure 8:**
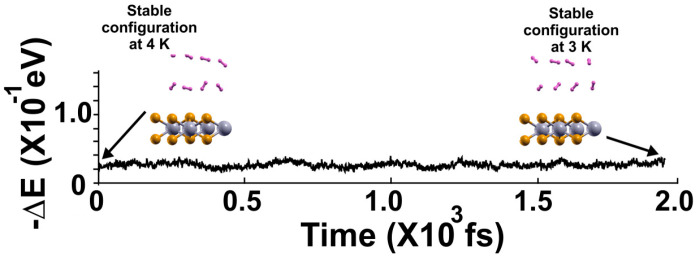
We performed the FPMD calculation at 3 K, taking the initial configuration of the steady system configuration as obtained at 4 K. The system reached a steady configuration after two picoseconds. Notice that at 3 K, the eight hydrogen molecules moved back to the surface, indicating that they adsorbed on the pristine 2D MoP_2_ surface at 4 K. Thus, the total adsorbed molecules are sixteen, so *n* = 16 in Equation (2). We obtained a gravimetric capacity for molecular hydrogen storage of 5.11% at 4 K.

In the following FPMD calculation at 3 K, the system reaches a steady configuration after two picoseconds. We can see that the eight hydrogen molecules in the steady configuration at 4 K moved back to the surface at 3 K, indicating that they were adsorbed on the pristine 2D MoP_2_ surface at 4 K, as Figure 8 shows. Thus, we have the adsorption of eight molecules on each face of the material for a total of sixteen adsorbed hydrogen molecules at 4 K. Thus, *n* = 16, and the gravimetric capacity from Equation (2) is 5.11% at 4 K.

In summary, in the optimization, the pristine 2D MoP_2_ surface adsorbed up to sixteen hydrogen molecules (eight per face of the material), with a gravimetric molecular hydrogen storage capacity of 5.11% and an average adsorption energy of −0.13 eV/molecule. However, we performed an FPMD calculation at 300 K and applied our already mentioned criterion to determine how many molecules adsorbed the surface at a given temperature. At 300 K, we found that only two (per face of the material) hydrogen molecules adsorbed on the pristine 2D MoP_2_ surface, with a gravimetric capacity of 1.28%. When we considered a temperature of 77 K and applied the same criterion, we found a gravimetric capacity for molecular hydrogen storage of 1.92%. For 4 K, the gravimetric capacity increases to 5.11%.

### 2.2. Hydrogen Storage on 2DMoP_2_ with Mo Vacancies

#### 2.2.1. Optimization

**Figure 9:**
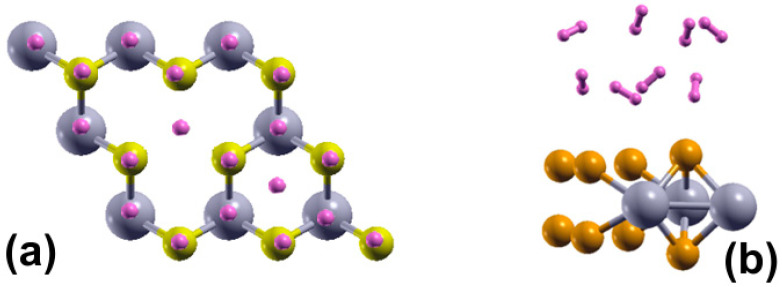
In (**a**), we show the system’s initial configuration from above; the supercell has a Mo vacancy and a total of three Mo atoms instead of four. We added hydrogen molecules one by one above a Mo (or Mo vacancy) or P atom, and the original distance of the hydrogen molecules from the surface was 3 Å before optimizing the system. In (**b**), we have the optimized configuration of the 2DMoP_2_ surface with a Mo vacancy with eight adsorbed hydrogen molecules. This occurs on each face of the 2D material. Thus, the surface adsorbed sixteen molecules, which means *n* = 16 in Equation (2), and a gravimetric capacity of 6.022%. The average adsorption energy is −0.14 eV/molecule, a little more potent than on the pristine surface.

To simulate the adsorption of H_2_ molecules on the surface of 2D MoP_2_ with vacancies, we considered a supercell 2 × 2 × 1, as Figure 1b shows. In this manner, we made a Mo vacancy in the cell of twelve atoms, corresponding to 12.5% of vacancies. Again, in the system’s initial configuration, we added hydrogen molecules one by one above a Mo or P atom (or Mo vacancy) before optimizing the system, and the original distance of the hydrogen molecules from the surface was 3 Å. We obtained that 2D MoP_2_ with Mo vacancies can adsorb up to sixteen H_2_ molecules (eight molecules per face of the material), as Figure 9 shows. Using Equation (1), we obtained an average adsorption energy of −0.14 eV/molecule, slightly more robust than on the pristine surface. From Equation (2), with *n* = 16, we got a gravimetric capacity of 6.022%.

#### 2.2.2. First-Principles Molecular Dynamics Calculations

**Figure 10:**
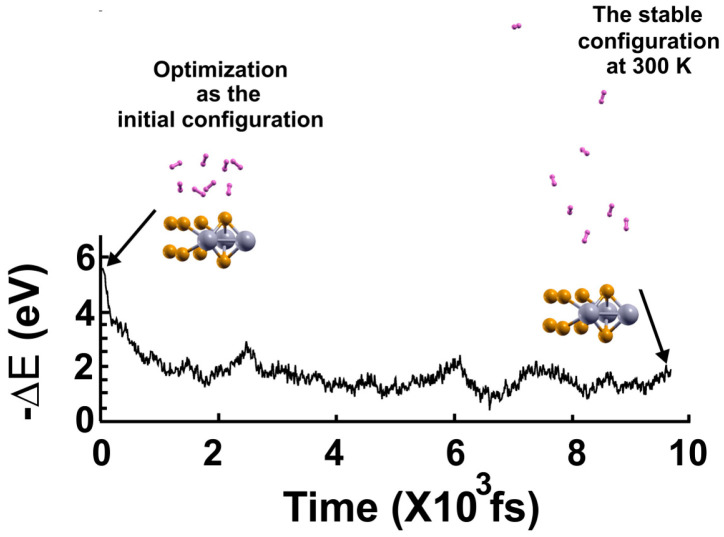
The FPMD calculation is at 300 K, with the optimization as the initial configuration. The supercell has a Mo vacancy and three Mo atoms instead of four. After ten picoseconds, the system reached a steady configuration, oscillating around the same value in −∆E. The eight H_2_ molecules are displaced away from the surface. Afterward, we diminished the system’s temperature from 300 K to 3 K to determine how many molecules remained bound to the surface at 300 K, as shown in Figure 11, to calculate the gravimetric capacity at 300 K from Equation (2).

**Figure 11:**
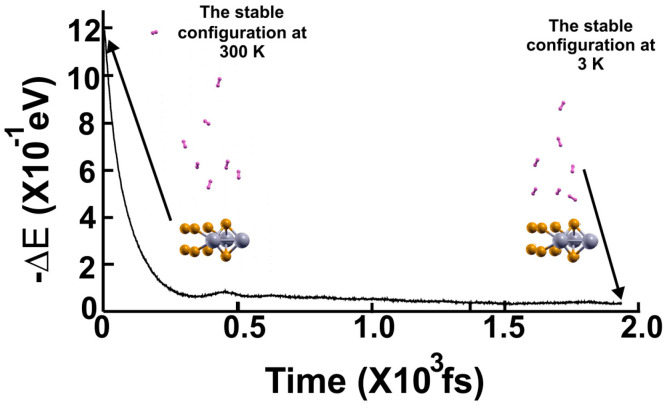
FPMD calculation at 3 K, where the initial state is the steady configuration at 300 K of the 2D MoP_2_ with Mo vacancies. The system reaches a steady configuration at around two fs. Notice that seven hydrogen molecules got closer to the surface, indicating seven remained adsorbed on the pristine 2D MoP2 surface at 300 K. We identified as adsorbed H_2_ molecules, only those that moved back to the surface. In this way, at 300 K, the surface with Mo vacancies retained seven H_2_ molecules per face of the 2D material, 14. Thus, using *n* = 14 in Equation (2), we obtained a gravimetric storage capacity of 5.27% at 300 K.

We followed the procedure in Section 4 to calculate the gravimetric capacity at 300 k. Figure 10 shows the FPMD calculation at that temperature, with the optimization as the initial configuration. The supercell has a Mo vacancy and three Mo atoms instead of four. After ten picoseconds, the system reached a steady configuration. As expected, all the molecules displaced away from the surface. Then, we diminished the system’s temperature from 300 K to 3 K to determine how many molecules were bound to the surface at 300 K, see Figure 11, to calculate the gravimetric capacity at 300 K using Equation (2).

Figure 11 shows the subsequent FPMD calculation at 3 K, starting with the steady configuration at 300 K. After reaching a steady configuration (at ~two fs), we can see that seven hydrogen molecules got closer to the surface. The remaining one did not change the distance from the surface it had at 300 K, indicating seven remained adsorbed on the pristine 2D MoP_2_ surface at 300 K. We identified as adsorbed H_2_ molecules, only those that moved back to the surface. In this way, at 300 K, the surface with Mo vacancies retained seven H_2_ molecules per face of the 2D material, 14. Thus, using *n* = 14 in Equation (2), we obtained a gravimetric storage capacity of 5.27% at 300 K.

**Figure 12:**
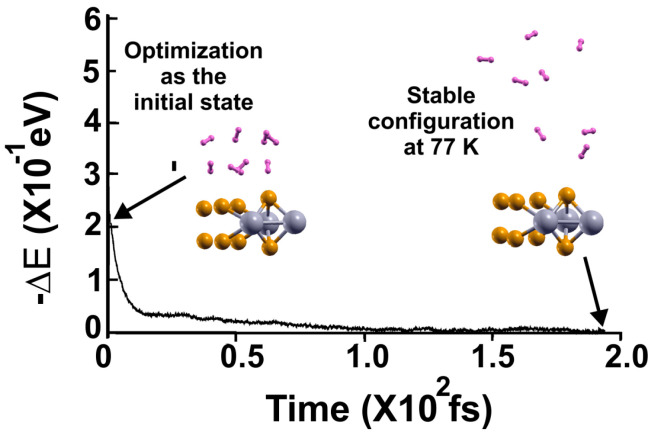
FPMD calculation at 77 K, where the initial state is the optimization. After 200 femtoseconds, the system reached a steady configuration at 77 K. All the molecules displaced away from the surface. To find the gravimetric capacity at 77 K, we must determine how many molecules remained adsorbed to the surface at that temperature. Thus, we performed a subsequent FPMD calculation for a much lower temperature, at 3 K, as Figure 13 shows.

**Figure 13:**
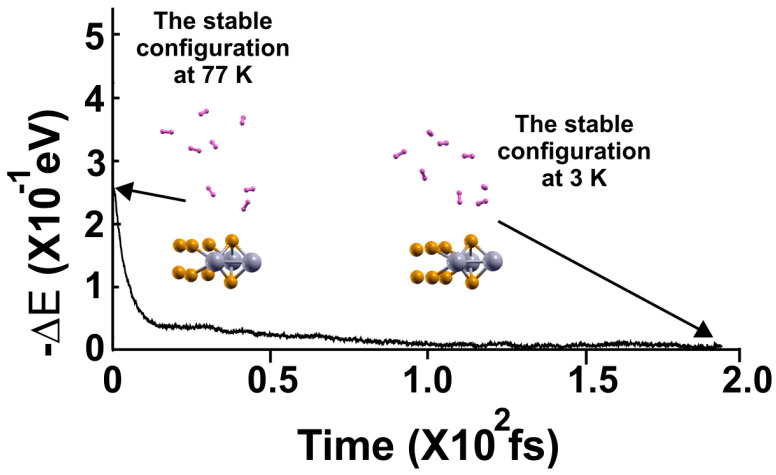
FPMD calculation at 3 K, where the initial state is the steady configuration at 77 K of the 2D MoP_2_ with Mo vacancies. The system reached a steady configuration after 200 femtoseconds. We can notice that all the hydrogen molecules moved back toward the surface. Thus, the eight molecules per face of the 2D material remained attached to the surface; this corresponds to *n* = 16 in Equation (2). We got a gravimetric capacity for molecular hydrogen storage of 6.02% for 77 K.

In the case of 77 K, we again considered the optimization configuration as the initial state and performed an FPMD calculation at that temperature, as Figure 12 shows. The system reaches a steady configuration at 77 K after 200 femtoseconds. Again, all the molecules moved away from the surface. After considering that temperature, we must determine how many molecules remained adsorbed to the surface. Thus, we proceeded to the subsequent FPMD calculation at 3 K, starting with the steady configuration at 77 K, as shown in Figure 13.

In Figure 13, we present the subsequent FPMD calculation at 3 K, starting with the steady system configuration at 77 K. Notice that all the eight hydrogen molecules (per face of the 2D material) moved towards the surface when the temperature decreased to 3 K. This indicates that at 77 K, we have sixteen adsorbed molecules. Thus, *n* = 16 in Equation (2), and we concluded that 77 K we obtained a gravimetric capacity of 6.02%.

**Figure 14:**
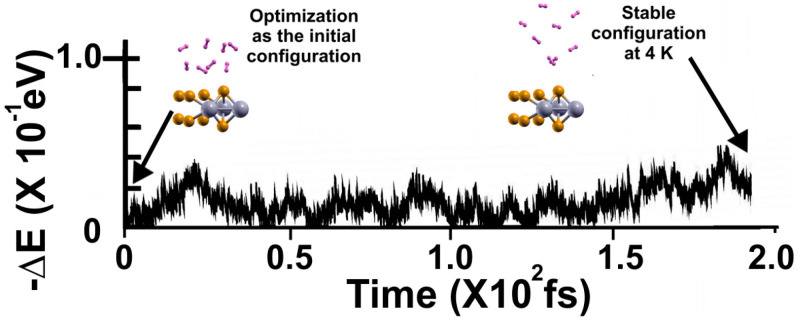
FPMD calculation at 4 K, where the initial state is the optimization. The system reached a steady configuration after 200 femtoseconds. All the molecules moved away from the surface. However, they remained much closer to the surface than in the other cases, 300 K and 77 K. To obtain the gravimetric capacity, we must determine how many hydrogen molecules remained adsorbed on the surface. Thus, we performed another FPMD calculation at 3 K, as shown in Figure 15.

**Figure 15:**
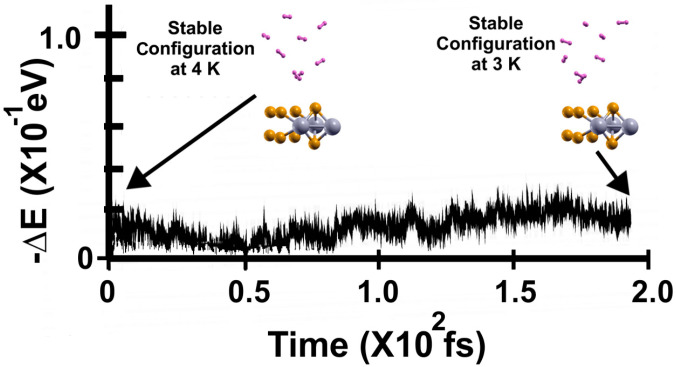
FPMD calculation at 3 K, where the initial state is the steady configuration at 4 K of the 2D MoP_2_ with Mo vacancies. The system reached a steady configuration at around 200 femtoseconds. Notice that all eight hydrogen molecules (per face of the 2D material) moved toward the surface when the temperature decreased to 3 K. Thus, the eight hydrogen molecules (per face) remained attached to the surface. Therefore, *n* = 16 in Equation (2), and the gravimetric capacity is 6.02% at 4 K.

Figure 14 shows the FPMD calculation at 4 K, taking the optimization as the initial configuration. After 200 femtoseconds, the system reached a steady configuration. Notice that all the molecules remained much closer to the surface than in the other two cases. Still, the molecules are displaced away from the surface. To know how many molecules were adsorbed at 4 K, we performed a second FPMD calculation at 3 K, as shown in Figure 15, taking the steady configuration obtained at 4 K as the initial configuration.

In Figure 15, we present the subsequent FPMD calculation at 3 K, starting with the steady configuration at 4 K. The system reached a steady configuration at around 200 femtoseconds. Notice that all eight hydrogen molecules (per face of the 2D material) moved toward the surface when the temperature decreased to 3 K. Thus, the sixteen molecules remained adsorbed on the surface, and *n* = 16 in Equation (2), showing a gravimetric capacity of 6.02% at 4 K.

In summary, when we considered the 2D MoP_2_ with Mo vacancies and optimized the system, the surface adsorbed up to 16 hydrogen molecules (8 per face of the 2D material). This corresponds to a gravimetric molecular hydrogen storage capacity of 6.02%, and the average adsorption energy is −0.14 eV/molecule. However, when we applied our already described approach to determine how many molecules adsorbed the surface at a given temperature and performed an FPMD calculation at 300 K, we found that only seven (per face of the 2D material) hydrogen molecules adsorbed on the surface. The gravimetric capacity is 5.27%. Using the same methodology at 77 K, we found a gravimetric capacity for molecular hydrogen storage of 6.02%. Finally, and as we expected, for 4 K, the gravimetric capacity is 6.02%, too.

## 3. Discussion

Given that currently, the first-principle calculations applied in the exploration of new materials for hydrogen storage are performed by solely simple energy optimization (see, for example, [34]), we propose a criterion using a first-principle molecular dynamics approach to test the total attraction forces on the physisorbed molecule. We performed ab initio DFT calculations. We applied it to investigate the hydrogen storage on the 2D material MoP_2_ at different temperatures. However, this method has a general application for the physisorption of molecules on any surface. We considered the 2D material pristine and with 12.5% of Mo vacancies.

Our investigation aims to predict whether the molecular physisorption obtained by simple energy optimization occurs for a given temperature. This means determining whether the kinetic energy given to the system destroys the molecular physisorption obtained by simple energy optimization when the effect of temperature is included. For this purpose, we utilized FPMD calculations.

We found by optimization of the system that the pristine 2D MoP_2_ surface adsorbed a maximum of 16 hydrogen molecules (8 per face of the 2D material), with an average adsorption energy of −0.13 eV/molecule. Furthermore, we considered the same surface with 12.5% Mo vacancies and obtained by optimization a gravimetric capacity of 6.02% and an average adsorption energy of −0.14 eV/molecule. We had to consider dispersion forces. Thus, we included the van der Waals interactions using Grimme’s semiempirical approach [14].

Then, we used our method to determine if the hydrogen physisorption obtained by simple energy optimization is possible for a given temperature. First, we performed an FPMD calculation at temperature T_1_ using the optimized system configuration as the initial setup for a long enough time (~1 ps) to obtain a steady system configuration at that temperature T_1_. Then, we conducted a second FPMD calculation at a lower temperature, T_2_ (where T_2_ << T_1_), using the final steady configuration from the first FPMD calculation as the initial setup. If total attraction forces were on the molecules at T_1_, they would return to the surface when the temperature reduces to T_2_ because of kinetic energy reduction. We counted as adsorbed hydrogen molecules at temperature T_1_, only those that moved back toward the surface at the lower temperature.

We followed the criterion described above to calculate the gravimetric storage capacity for molecular hydrogen storage for 300 K, 77 K, and 4 K. Our results suggest that using only optimization may lead to unreliable predictions for hydrogen storage. We conclude that the 2D MoP_2_ surface with Mo vacancies was more adequate than the pristine surface. At 300 K, the defective 2D MoP_2_ with 12.5% Mo vacancies had a 5.27% gravimetric capacity (the US-DOE target is 5.5% for automotive-grade molecular hydrogen storage). At 77 K and below this temperature, this system reached a gravimetric capacity of 6.02%, above the US-DOE target. Here, the disadvantage is maintaining the material at that temperature during the automotive-grade application for hydrogen storage.

While maintaining the material at the required temperature is challenging, our results indicate that further investigation of this class of 2D materials could lead to successful molecular hydrogen storage at room temperature and ambient pressure.

## 4. Materials and Methods

We performed ab initio DFT calculations (see Appendix A) to study hydrogen storage on the 2D material MoP_2_. We considered the pristine material and the same material with 12.5% Mo vacancies. We employed the generalized gradient approximation (GGA) with the exchange-correlation energy given by Perdew–Burke–Ernzerhof double-zeta polarized basis sets and norm-conserving pseudopotentials [40,41]. Our calculations used the SIESTA code [42] and the spin-polarized density functional formalism. We followed the Monkhorst–Pack scheme [43] and considered a Brillouin zone sampling of 24 × 24 × 1 k-points. We took an energy cutoff of 180 Ry for numerical integrations. The geometries of the studied systems converged until forces between atoms were smaller than 0.01 eV/Å. To consider dispersion forces, we included the van der Waals interactions using Grimme’s semiempirical approach [44,45].

Every structure was relaxed to determine the most stable configuration before the saturation of H_2_ molecules. We calculated the adsorption energy using Equation (1).

(1) $$E_{\mathrm{ads}}=E_{\mathrm{final}\;\mathrm{configuration}}\;–\;\left(nE_{H}+E_{\mathrm{surface}}\right),$$

Here, *E_ads_* is the adsorption energy, *n* is the number of adsorbed hydrogen molecules, *E_H_* is the total energy of one free H_2_ molecule, and *E_surface_* is the total energy of the surface supercell. We calculated the gravimetric capacity *Wt*% using Equation (2).

(2) $$\mathrm{Wt}\%=100\;nW_{H}/\left(nW_{H}+W_{\mathrm{surface}}\right),$$

where *W_H_* is the molecular hydrogen mass, *n* is the same as before, and *W_surface_* is the mass in the surface supercell. We have 4 Mo atoms (or three if we have a Mo vacancy) and 8 P atoms in the supercell. Thus, *W_surface_* = 4 *W_Mo_* + 8 *W_P_* (for the pristine surface) or *W_surface_* = 3 *W_Mo_* + 8 *W_P_* (for a surface with a Mo vacancy). We have *W_Mo_* = 95.950; *W_P_* = 30.974; *W_H_* = 2.016.

When optimizing the energy of a system, we can make accurate predictions for the adsorption energy when there is a strong attraction, such as in chemisorption. However, for physisorption processes with energies below 0.2 eV, including London dispersion forces (Van der Waals interactions), it is necessary to increase the accuracy of the adsorption energy calculation, which must be much more significant. Furthermore, the system’s temperature and pressure can influence the adsorption energy. Hydrogen adsorption changes in the solid-state hydrogen storage when we have different temperature and pressure values. For example, there are reports of hydrogen storage at pressures of 10 bar, 15 bar, and 50 bar, and temperatures of 77 K and 300 K [43]. According to US DOE recommendations for storing hydrogen, it is necessary to have adsorption energies between 0.1 eV and 0.2 eV for effective adsorption and desorption processes at room temperature and pressure around some atmospheres [2,3]. This range of molecular hydrogen binding energy values is between physisorption and chemisorption phenomena. Thus, as mentioned above, we had to include van der Waals interactions. We used Grimme’s semiempirical approach [44,45]. The DFT-D method by Grimme is a semiempirical correction to DFT for considering the London dispersive interactions, and it is based on damped, atomic-pairwise potentials. The basic idea is to replace part of the nonlocal, long- and medium-range electron correlation effects in a conventional gradient corrected density functional by damped (~1/(R^6^) dependent terms. It is a low-cost approach like semiempirical models. However, the DFT-D method is sound. It has been tested thoroughly and applied successfully on numerous kinds of systems. The technique has been used successfully in surface science and solid state [46,47,48,49] and has a lower cost than the D3 [50] and D4 [51] approaches.

We used FPMD calculations to include the effect of temperature and pressure on the binding forces on the hydrogen molecules.

Furthermore, following US DOE recommendations, the hydrogen storage capacity should be six weight percent. Finding the right solution is challenging because hydrogen molecules may be too strongly or weakly adsorbed in light materials [3]. We chose a 2D MoP2 surface that is pristine or with Mo vacancies as a possibility.

We have developed a criterion to test the total attractive forces in hydrogen storage at a specific temperature. With this approach, we predict whether the molecular physisorption obtained by simple energy optimization can exist for a given temperature.

The van der Walls forces occur when we have surface and molecule polarization processes. The molecule polarization will depend on the average distance between its atoms, which will depend on the temperature; additionally, this polarization depends on the distance of the molecule from the surface. The closer the molecule, the more intense the polarization and the force on the molecule. Additionally, the distance of the molecule from the surface will depend on its kinetic energy, i.e., on the temperature. Furthermore, when we perform FPMD calculations, the temperature is included, and the kinetic energy of the atoms on the surface and of the molecule’s atoms are considered. This kinetic energy consists of each molecule’s vibrations, rotations, velocities, and interactions. Of course, this is for every particle of the whole system. The polarization process is more dynamic and temperature-dependent. Thus, the magnitude of the total attraction force on the molecule will depend on the temperature.

If, at some temperature value, the molecule has sufficient kinetic energy to be far enough from the surface and overcome energy barriers of the systems’ potential energy, it will escape from the surface bonding. The molecule will not be under the influence of the attraction force from the surface. Thus, it will not remain bound at a lower temperature. Similarly, if, at some temperature, the molecule does not have sufficient kinetic energy to be far enough from the surface and overcome the energy barriers of the systems’ potential energy, the molecule will be under the influence of the attraction force from the surface. If the temperature is decreased, the molecule will lose kinetic energy and get closer to the surface.

We first investigated the pristine surface and optimized the system formed with the surface and hydrogen molecules. This optimization leads to a value for the gravimetric capacity for hydrogen storage and the adsorption energy per molecule. Afterward, we used a first-principles molecular dynamics approach at various temperatures. To perform this calculation, it is necessary to establish a value for the temperature and hydrostatic pressure. We chose ambient temperature and pressure to compare our results with the DOE’s requirements for hydrogen storage. We conducted the following procedure to determine the number of molecules attached to the surface at the temperature T_1_.

We start performing an FPMD calculation at temperature T_1_ using the optimized system configuration as the initial setup for a long enough time to obtain a steady system configuration (for our case, ~1 ps) at that temperature T_1_. Then, we conducted a second FPMD calculation at a lower temperature, T_2_ (where T_2_ << T_1_), using the final steady configuration from the first FPMD calculation as the initial setup. If total attraction forces were on the molecules at T_1_, they would return to the surface when the temperature reduces to T_2_ because of kinetic energy reduction. We counted as adsorbed hydrogen molecules at temperature T_1_, only those that moved back toward the surface at the reduced temperature. In our FPMD calculations, we assumed an isolated system. It cannot exchange energy or particles with its environment, so its total energy does not change with time. We considered a microcanonical ensemble, so we chose the NVE ensemble. The macroscopic variables of the ensemble are the total number of particles in the system (N), the system’s volume (V), and the total energy in the system (E). For the temperature control, we used velocity rescaling. We followed the parallelizing workload given in the Quantum Espresso code [51,52], and we used 32 processors, and the total employed computer time was around nine weeks. For visualization of the different surfaces and configurations, we used the XCrysen code [53].

## Data Availability

Data are contained within the article.

## Appendix A

The foundational work of the DFT formalism is given in 1964 and 1965 in two articles [54,55].

Walter Kohn won the Nobel Prize in Chemistry 1998 “for developing the density-functional theory”. He shared this award with John Pople.

DFT is based on quantum theory and does not require any empirical parameter. The only input data are the atomic number of the atoms’ system and some structural information. The many-body problem of interacting electrons is replaced by an equivalent one-electron problem, where each electron moves under an effective potential. It has been successfully applied to various solids’ structural or dynamical properties (lattice structure, charge density, magnetization, phonon spectra, etc.). DFT is now by far the most widely used electronic structure method. It has been utilized since the beginning of FPMD calculations. It is an extension of the ideas contained in the theory developed independently by Llewellyn Thomas and Enrico Fermi., where the atomic calculations do not require a wave function. Furthermore, the electron density was the fundamental variable in Thomas–Fermi’s (T.F.) theory in 1927.

In their work of 1964, Hohenberg and Kohn [55] proved that total electronic energy, as well as other properties, is a functional of the electron density. They also proved that the exact ground state electron density into the universal functional yields the global minimum value of the energy functional. They also proved that the universal density functional exists. Their work did not prescribe the form for this density functional or how the electron density should be described. The equations developed by Kohn and Sham made DFT a practical computational tool. The Kohn–Sham equation is the Schrödinger equation of a virtual system of noninteracting electrons that generate the same density as the system of interest. But it includes, in principle, precisely the many-body effects through a local exchange-correlation potential whose exact form is unknown.

The electron density can be represented using several different types of mathematical functions. The original DFT calculations utilized plane-wave functions as basis sets. The infinite extent of these functions was ideal for numerical simulations on extended structures such as metal surfaces or crystalline solids. Atom-centered basis sets are generally more appropriate for calculations of discrete atoms and molecules. Thus, DFT is computationally much less expensive than the traditional ab initio multiple electron wave function approaches.

The Kohn–Sham equations [55] are the following.

In the 1964work they proved that the ground-state energy of an interacting inhomogeneous electron gas with density *n*(***r***) in a static potential *V*(***r***) is

(A1) $$E=\int V\left(r\right)n\left(r\right)dv+\frac{1}{2}\int \frac{n\left(r\right)n\left(r^{\prime }\right)dv\;dv^{\prime }}{\left|r-r^{\prime }\right|}+G\left[n\right]\;$$

(A2) $$G\left[n\right]=T_{s}\left[n\right]+E_{xc}\left[n\right]$$

(A3) $$T_{s}\left[n\right]=\frac{1}{2}\sum_{i=1}^{N}\int \left[\nabla^{2}\varphi_{i}(r)\right]dv\;\;\;\;,$$

where *Ts*[*n*] is the kinetic energy and *Exc*[*n*] is the exchange and correlation energy of an interacting inhomogeneous electron gas with density *n*(***r***). For an arbitrary *n*(***r***), having a simple expression for *Exc*[*n*] is impossible. Furthermore, *n*(***r***) is obtained from the equation:

(A4) $$n\left(r\right)=\sum_{i=1}^{N}{\left|\varphi_{i}\left(r\right)\right|}^{2},$$

where *N* is the number of electrons, the wave function *φ_i_*(***r***) is the solution of the one particle Schrödinger’s equation:

(A5) $$-\frac{1}{2}\nabla^{2}\varphi_{i}\left(r\right)+\left[V_{eff}\left(r\right)\right]\varphi_{i}\left(r\right)=\in_{i}\varphi_{i}\left(r\right)\;\;\;,$$

(A6) $$V_{eff}\left(r\right)=V\left(r\right)+\int \frac{n\left(r^{\prime }\right)dv^{\prime }}{\left|r-r^{\prime }\right|}+\mu_{exc}\left(r\right)\;\;,$$

With

(A7) $$\mu_{exc}\left(r\right)=\frac{{\delta E}_{xc}\left[n\right]}{\delta n(r)}\;\;,$$

notice that $\mu_{exc}(r)$ is a functional derivative. It is possible to find many approximations of *Exc*[*n*] in the literature.

The wave function $\varphi_{i}(r)$ is not the real electron wave function. However, the expression for the electron density corresponds to the real solution. Equations (A1)–(A7) must be solved numerically in a self-consistent way. It is possible to start with a proposed *n*(***r***) and then obtain *V_eff_* (***r***) from Equations (A6) and (A7). It is necessary to have an approximate expression to *Exc*[*n*]. Afterward, to obtain *n*(***r***), use Equations (A4) and (A5) to compare with the initial *n*(***r***). Having the self-consistent solution for *n*(***r***), the energy is calculated from Equation (A1), and all the properties of the system under study are obtained.
